# Reproducibility of isokinetic knee testing using the novel isokinetic SMM iMoment dynamometer

**DOI:** 10.1371/journal.pone.0237842

**Published:** 2020-08-31

**Authors:** Tim Kambič, Mitja Lainščak, Vedran Hadžić

**Affiliations:** 1 Department of Research and Education, General Hospital Murska Sobota, Murska Sobota, Slovenia; 2 Laboratory of Sports and Medical Diagnostics, Faculty of Sport, University of Ljubljana, Ljubljana, Slovenia; 3 Division of Cardiology, General Hospital Murska Sobota, Murska Sobota, Slovenia; 4 Faculty of Medicine, University of Ljubljana, Ljubljana, Slovenia; Texas State University, UNITED STATES

## Abstract

Isokinetic dynamometry is the gold standard for testing maximal strength in elite sport and rehabilitation settings. To be clinically useful, such tests should be valid and reliable. Despite some evidence regarding the relative test vs retest reliability of knee dynamometry, there is still a paucity of research regarding the absolute reliability parameters. The purpose of this study was to assess the absolute and relative intra-device reproducibility of isokinetic knee flexion and extension using the novel SMM iMoment dynamometer. A total of 19 participants (13 males and 6 females, aged 24 (2) years, height 178 (9) cm and weight 76 (11) kg) performed two identical knee isokinetic tests with at least a week of rest between measurements. Peak torque of knee extension and flexion were determined at 60°/s. Moderate (0.892) to excellent (0.988) relative reliability using the intraclass correlation coefficient (ICC) was obtained for peak knee torque. Absolute reliability assessed with a standard error of measurement (SEM %) was low, ranging from 2.54% to 6.93%, whereas the smallest real difference (SRD %) was moderate, ranging from 7.04% to 19.22%. Furthermore, there were no significant correlations between means and differences of two measurements, and Bland-Altman plots also showed no signs of heteroscedasticity. Our measurement protocol established the moderate to excellent reliability of the novel SMM iMoment isokinetic dynamometer. Therefore, this dynamometer can be applied in sport rehabilitation settings to measure maximal knee strength.

## Introduction

Voluntary muscle contraction is vital for human physical functioning [1], as muscles generate joint forces necessary for movement, joint stabilization, and posture maintenance [2]. Thus, the accurate assessment of individual’s muscular capacities is important to identify possible weakness related to disease or ageing [2], and later appropriately prescribe and monitor the progress of the athletic or rehabilitation exercise program [3].

Muscle strength can be expressed in numerous ways, including maximum weight lifted on an exercise device, maximum isometric and maximum isokinetic torque with angle specific or nonspecific assessment [1]. Since the early introduction of isokinetic dynamometry in 1967 [4], the method has become the gold standard in the evaluation of muscle performance and pathology in research, elite sport, and clinical practice [5,6]. An isokinetic dynamometer assesses joint-related muscle maximal concentric and eccentric strength under constant velocities throughout the range of motion [7]. To be clinically significant, such tests should be valid and reliable. The test-retest reliability was previously assessed using different isokinetic machines, such as Biodex [8–10], Cybex [6,11–14], Kin Com [5,15], Merac [16], Lido [17], iSam 9000 [18], and Technogym’s REV9000 [7,19].

The majority of the older studies evaluated test-retest reliability of knee isokinetic torque only using the intraclass correlation coefficient (ICC) as an indicator of relative reliability. Some older studies using the ICC showed excellent reliability (>0.92) [8,11,13,20,21], while others, mainly more recent studies all demonstrated good (0.89) to excellent (0.98) test-retest reliability of knee flexors and extensors at a velocity 60 °/s [5,7,9,14,22,23]. In contrast, only a few studies have also examined the absolute reliability using the standard error of measurement (SEM) and/or the smallest real difference (SRD) for knee peak torque and work [5,7,14,19,21]. The SEM and SRD varied between 3.5–6.7% and 9.7–19.47%, respectively [5,7,14,21].

Furthermore, there is a paucity of research regarding the SRD during eccentric knee flexion, with only three studies published [5,7,14]. In addition, most of the previous dynamometers settings were controlled manually, from the repositioning of the dynamometer axis to changes of seat settings and starting position angle of the arm or leg [5,7,13,14,16,18,19,21,22]. To date, no device has used software manoeuvred robotic dynamometer position adjustments, which would have the potential to improve the reliability of the measurement of isokinetic maximal knee torque further. Therefore, this study aimed to determine the test-retest absolute and relative reliability of knee peak torque flexion and extension on the novel iMoment dynamometer, and to encourage its possible use in clinical and research settings.

## Methods

### Study design

This study was designed as a reproducibility study in a test-retest fashion, with at least one week break between both tests, as advised by the previous studies on the reliability of isokinetic testing [5]. Both tests were conducted during the same time of the day to reduce the effect of diurnal subject-linked variability [5]. To additionally optimize the accuracy of the measurements, all tests were conducted by the same researcher (TK). The leg testing order was randomly selected (e.g., leg tested first on the test, was tested second on a retest) in order to minimize the possible learning effect. The absolute and relative reliability was assessed with SEM (%), SRD (%) and ICC, respectively.

### Subjects

Out of 24 healthy recreational subjects initially enrolled in the study, 19 completed both isokinetic tests (13 males and 6 females, aged 24(3) years, height 178 (9) cm, weight 76 (11) kg, all were left leg dominant (100%)) with 8 (3) days break between measurements. The dominant leg was defined as the leg used to kick a ball [24]. Four participants were excluded due to health problems (pain during or after the trial repetitions and/or test) and one participant left the study due to personal reasons.

No adverse cardiovascular or musculoskeletal problems were reported during the data collection. All subjects were advised to continue with their normal physical activity regimen, with the exception of vigorous intensity aerobic activities and sports, and lower limb strength training during the study. In addition, subjects were advised to avoid any moderate to vigorous physical activities at least two days prior to measurements and verbally recalled all recent physical activities to the researcher to ensure similar pre-test conditions.

Prior to inclusion into the study, all participants were informed about the methods and procedures, as well as possible risks during the isokinetic testing. Written consent was signed prior to inclusion into the study. The study was conducted according to the Declaration of Helsinki guidelines for the use of human participants. The study protocol was approved by the Board of Ethics in Sport, held at the Faculty of Sport, University of Ljubljana (identifier: 15/2018).

### Study protocol

**Fig 1:**
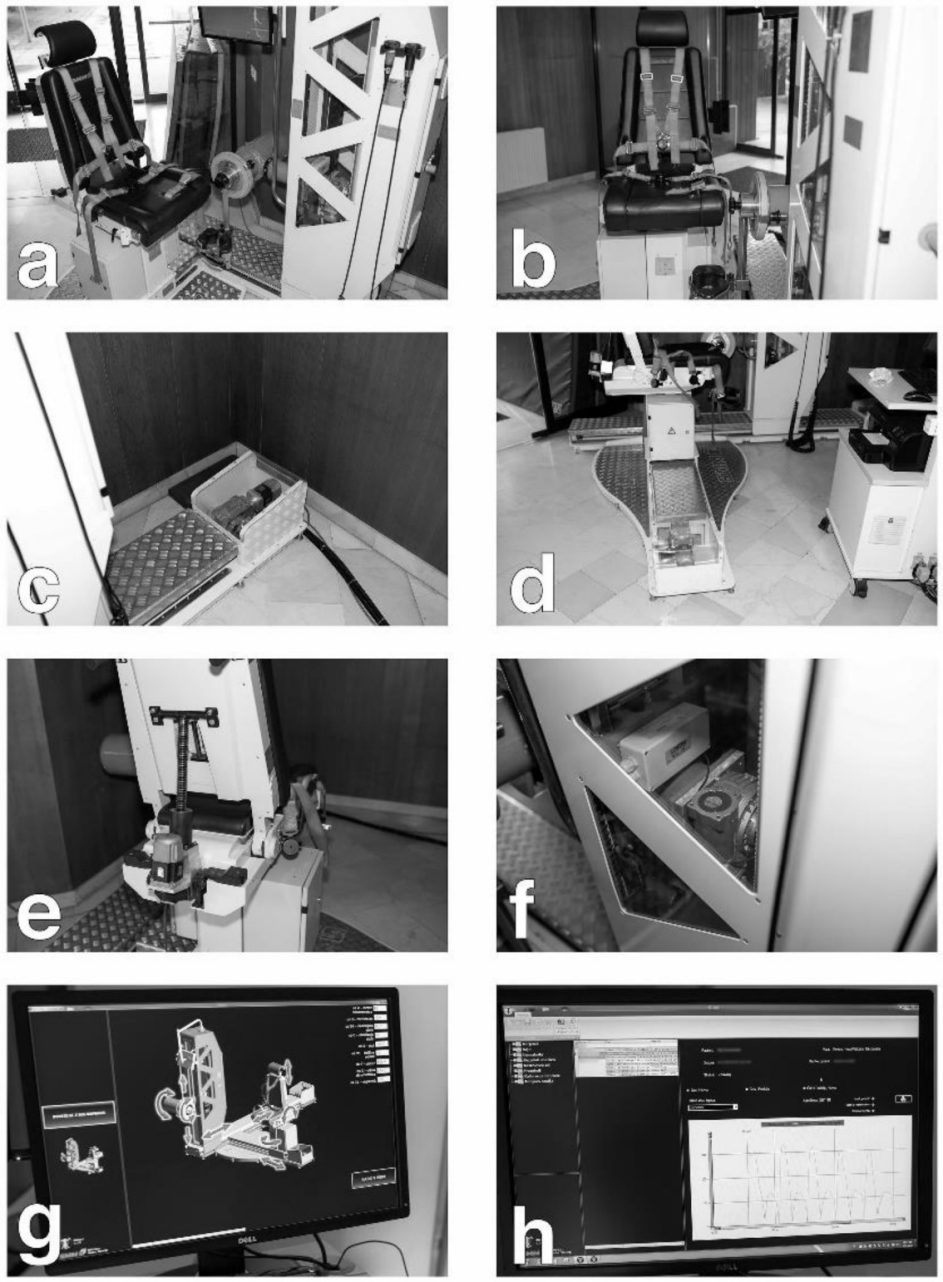
The novel SMM isokinetic dynamometer.

Measurements were performed on an isokinetic dynamometer SMM iMoment (SMM production systems, Ltd., Maribor, Slovenia) using a standard leg attachment. This is a novel self-constructed dynamometer in co-operation between SMM d.o.o., Faculty of Sport in Ljubljana and Faculty of Mechanical Engineering in Ljubljana (Fig 1). The device is a robotic dynamometer that is operated through software in all aspects, including dynamometer height, dynamometer position, chair position, seat length, seat backrest inclination as well as the rotation of the chair. Prior to each testing day, the machine was calibrated using a standard calibration weight (31.3 Nm), and before each test, the participant’s leg was weighted for gravitational error torque (GET).

The warm-up consisted out of 8 minutes of cycling at 100 to 120 W with a cadence of 60 to 70 cycles per minute. Later all subjects performed a short dynamic stretching of lower limb and 10 repetitions of squat and hip thrust exercise. The test was performed with the participants in the sitting position. Participants were strapped with belts across the chest, pelvis and test leg thigh to minimize body movement and compensations of other muscles. Furthermore, the dynamometer axis of rotation was aligned with knee’s joint axis of rotations using lateral epicondyle as an anatomic mark. The range of motion was 60° (from 90° to 30° of knee flexion, with full knee extension being 0°).

After the general warm-up, each subject performed 10 submaximal concentric contractions of knee extension and flexion and 5 submaximal eccentric contractions in flexion at 60 °/s as part of a special warm-up and familiarization with the test. Intensity in the general warm up sets was progressed during each repetition from 50% to 80% of individual`s perceived maximal strength of knee extensors and flexors. During the testing, subjects performed 5 maximal concentric contractions of knee extension and flexion in the first set followed by 5 maximal eccentric contractions of knee flexion in the second set. In the first set, each concentric contraction of knee extensors was followed by concentric contraction of knee flexors; in the second set, eccentric contraction was followed by concentric contraction of knee flexors. There was a 2-minute break between both sets. Participants were verbally encouraged by the investigator to give their maximal effort, and visual feedback was provided throughout the test on the dynamometer`s monitor (Fig 1a).

### Statistical analysis

The iMoment’s software evaluation report provided data for each mode of contraction and muscle for the left and right legs. The highest peak contraction torque of each set of both tests was used in the reliability analysis. All data were calculated using the IBM SPSS Software for Windows (version 21, SPSS Inc., Armonk, New York, USA) and MedCalc (MedCalc Software, Seoul, Republic of Korea).

Categorical variables are displayed as numbers and percentages, and numeric variables are presented as means and standard deviations. All numeric variables were first checked for normality of distribution with Shapiro-Wilk’s test. The differences between test and retest were assessed with repeated measure analysis of variance (ANOVA) for normally distributed variables and with Friedman’s test in case of asymmetrically distributed variables.

The agreement between measurements was assessed with the intraclass correlation coefficient (ICC), and with the 95% confidence interval (95% CI) for ICC. Values of the ICC are interpreted according to recent guidelines [25]. Absolute and relative measurement error were assessed with the standard error of measurement ($\text{SEM}=\text{SD}\times \sqrt{1-ICC}$) and with the SEM %, respectively [5]. The latter represents the limit for the smallest change that indicates a real improvement for a group of participants following a given intervention (e.g., exercise training). To calculate the smallest change that indicates a real improvement for a single participant, the absolute value and the percentage (%) of the smallest real difference (SRD) was used [26]. In contrast to SEM, the SEM % and SRD % are independent of the units of measurement.

The qualitative assessment of systematic changes between test and retest means was performed using Bland-Altman plots. These graphs can illustrate the possible issue of heteroscedasticity, which occurs when the test-retest difference increases as the mean of the value of both test decreases [27]. Additionally, the quantitative assessment of heteroscedasticity was calculated with the Pearson`s correlation coefficient or Spearman’s rank correlation coefficient for normally or asymmetrically distributed variables, respectively. The significance level was set at p-values <0.05.

## Results

Table 1 summarizes test and retest findings. There were no statistically significant differences between test and retest in all measured isokinetic torque parameters (Table 1).

**Table 1:** Peak torque on test and retest.

		Mean (SD)	95% CI for Mean	% difference test vs re-test	F	p
	**Concentric extension at 60°/s**					
Left leg	Test	282.44 (64.00)	251.59, 313.29	4%	2.579	0.167*
	Re-test	271.60 (55.02)	245.08, 298.12			
Right leg	Test	243.82 (55.02)	217.30, 270.34	1%	0.761	0.395
	Re-test	241.31 (58.06)	213.33, 269.30			
	**Concentric flexion at 60°/s**					
Left leg	Test	141.86 (30.18)	127.31, 156.40	1%	0.288	0.598
	Re-test	140.64 (32.63)	124.91, 156.37			
Right leg	Test	145.87 (32.99)	129.97, 161.77	-1%**	0.337	0.569
	Re-test	147.63 (31.13)	132.63, 162.64			
	**Eccentric flexion at 60°/s**					
Left leg	Test	168.72 (38.84)	150.00, 187.45	1%	0.183	0.674
	Re-test	167.10 (37.77)	148.90, 185.30			
Right leg	Test	180.76 (36.30)	163.26, 198.25	4%	1.316	0.359*
	Re-test	172.88 (42.06)	152.61, 193.16			

*—Friedman test,

**—minus sign indicates better performance on retest, SD-standard deviation, CI-confidence interval, F-test statistic

Table 2 presents the ICC, and the absolute and relative reliability statistics. The mean values of ICC for maximal peak concentric torque of the left and right quadriceps, the left and right hamstring, and the peak eccentric torque of the left hamstring showed excellent reliability, while the ICC for peak eccentric torque of the right hamstring showed only good reliability. Based on the 95% CI for ICC, the reliability was excellent for the peak concentric torque of the left and right quadriceps, and the left hamstring. Moreover, the 95% CI for ICC showed good to excellent reliability for peak concentric torque of the right hamstring and peak eccentric torque for the left hamstring. Additionally, the reliability according to 95% CI for ICC was moderate to excellent for peak eccentric torque of the right hamstring. All ICC were significant (p<0.001).

**Table 2:** Reproducibility measures of the isokinetic concentric and eccentric knee flexion and extension.

	ICC	95% CI for ICC	p	d (Nm)	CVSD	SEM (Nm)	SEM %	SRD	SRD %
Concentric left quadriceps at 60°/s (Nm)	0.967	[0.914, 0.987]	<0.001	10.84	1.7%	10.67	3.85%	29.57	10.67%
Concentric right quadriceps at 60°/s (Nm)	0.988	[0.967, 0.995]	<0.001	2.51	1.5%	6.16	2.54%	17.07	7.04%
Concentric left hamstring at 60°/s (Nm)	0.975	[0.935, 0.990]	<0.001	1.21	2.2%	4.91	3.47%	13.60	9.63%
Concentric right hamstring at 60°/s (Nm)	0.955	[0.884, 0.983]	<0.001	-1.76	2.5%	6.66	4.54%	18.45	12.58%
Eccentric left hamstring at 60°/s (Nm)	0.951	[0.873, 0.981]	<0.001	1.62	2.4%	8.28	4.93%	22.95	13.67%
Eccentric right hamstring at 60°/s (Nm)	0.892	[0.718, 0.958]	<0.001	7.87	2.8%	12.26	6.93%	33.99	19.22%

ICC-intraclass correlation coefficient, d- mean difference between test and retest, CI-confidence interval, CVSD-coefficient of variation of standard deviation, SEM-standard error of measurement, SRD-smallest real difference

The values of CVSD and SEM were low. The lowest CVSD was obtained for the peak concentric torque of the left quadriceps (1.7%), as the peak eccentric torque of the right hamstring had the highest CVSD (2.8%). Similarly, the low SEM % ranged from 2.54% to 6.93% for peak concentric torque of right quadriceps and peak eccentric torque for right hamstring, respectively.

**Fig 2:**
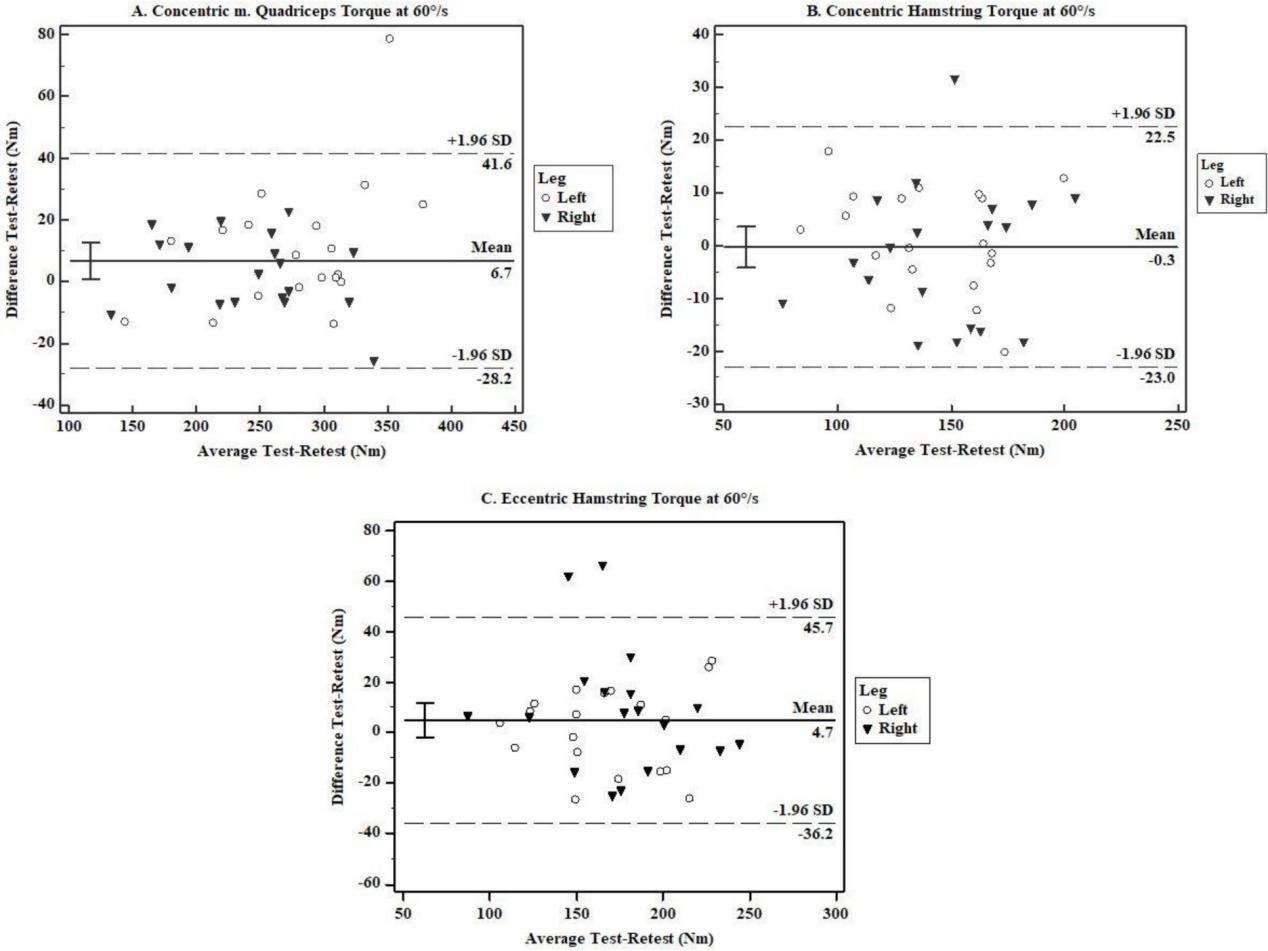
Test-retest qualitative agreement of concentric torque of Quadriceps (a.) and Hamstrings (b.), and eccentric torque of Hamstrings (c.).

Quantitative assessment of the systematic change showed no significant correlation between test-retest means and test-retest difference for all variables. All correlations values were low and ranged from -0.322 to +0.252. Furthermore, the qualitative results via the Bland-Altman plots showed good agreement between measurements and homoscedasticity for concentric torque of Quadriceps at 60°/s (-28.2; 41.6 Nm), concentric torque of the hamstring at 60°/s (-23.0;22.5 Nm) and eccentric torque of the hamstring at 60°/s (-36.2;45.7 Nm). We identified one outlier in concentric torque of the left quadriceps and the right hamstrings at 60°/s, and two outliers in the eccentric torque of the right hamstring (Fig 2a–2c).

## Discussion

The results have demonstrated an excellent test-retest reliability for concentric peak torque in knee extension and flexion, except for moderate reproducibility for the eccentric torque of the right hamstring. There were small test-retest differences and SRD was acceptable, indicating the potential for the clinical use of novel SMM iMoment dynamometer for the concentric and eccentric evaluation of knee flexion and extension.

The ICC values obtained in our study ranged from 0.892 (0.718–0.958) for the eccentric right hamstring peak torque to 0.988 (0.967–0.995) for concentric right quadriceps peak torque. This is in line with previously published data, which showed excellent reliability of knee extensors and flexors in a concentric and eccentric manner using both older [8,11,21] and the latest types of isokinetic dynamometers [5,14,22,23]. The absolute percentage difference between test and retest was low and varied between 1% and 4%. In addition, we have also performed a subsequent analysis to control for leg dominance and the leg tested first to eliminate any potential learning effects or effects of central fatigue, and the analysis have shown that the choice of first tested leg had no impact on the results.

Small measurement error and smallest real difference are important for clinical implication of the measurement protocol [5]. However, only a few previous studies reported the SEM, SEM %, and SRD values as indicators of absolute reliability [7,14,21] or real test-retest improvement of the sample [28] (Table 3). Our SEM values range (6.66–12.26 Nm) are in line with previous reports (5.57–13.00 Nm) using the Technogym’s REV 9000 dynamometer [19], the Kincom 500H dynamometer [5] or the IsoMed 2000 dynamometer [22].

**Table 3:** Overview of current studies examining absolute and relative reliability using different isokinetic dynamometers.

Device, year, reference	N	Type of contraction at 60°/s	ICC	SEM	SEM %	SRD	SRD %
Biodex, 1990, [8]	19	Concentric extension	0.95	NR	NR	NR	NR
		Concentric flexion	0.98	NR	NR	NR	NR
Cybex 6000, 1993, [11]	20	Concentric extension	0.94	NR	NR	NR	NR
		Concentric flexion	0.97	NR	NR	NR	NR
Cybex 6000 DYN, 1996, [13]	18	Concentric extension	0.84	NR	NR	NR	NR
		Concentric flexion	0.83	NR	NR	NR	NR
		Eccentric flexion	0.84	NR	NR	NR	NR
Biodex System 2, 1997, [21]	21	Concentric extension*	0.97	NR	4.8	NR	NR
		Concentric flexion*	0.97	NR	4.9	NR	NR
Biodex System 3 Pro, 2005, [9]	13	Concentric extension	0.98	NR	NR	NR	NR
		Concentric flexion	0.97	NR	NR	NR	NR
Tecnogym REV9000, 2006, [19]	16	Concentric right extension	0.89	10.68	8.52	NR	NR
		Concentric left extension	0.81	13.00	10.71	NR	NR
		Concentric right flexion	0.92	6.74	7.22	NR	NR
		Concentric left flexion	0.92	6.47	7.16	NR	NR
KinCom 500H, 2007, [5]	18	Concentric extension	0.93	8.21	6.48	22.75	17.95
		Concentric flexion	0.93	5.57	7.02	15.45	19.47
		Eccentric flexion	0.94	6.48	6.88	17.97	19.07
Cybex NORM, 2008, [14]	18	Concentric right extension	0.98	NR	4.3	NR	12.0
		Concentric left extension	0.95	NR	4.7	NR	13.0
		Concentric right flexion	0.95	NR	5.2	NR	14.5
		Concentric left flexion	0.93	NR	6.7	NR	18.6
		Eccentric right flexion	0.94	NR	6.5	NR	18.0
		Eccentric left flexion	0.97	NR	5.2	NR	14.5
IsoMed 2000, 2012, [22]	35	Concentric right extension	0.96	8.7	NR	NR	NR
Cybex II, 2013, [6]	16	Concentric extension	0.95	NR	NR	NR	NR
		Concentric flexion	0.89	NR	NR	NR	NR
Technogym REV9000, 2013, [7]	24	Concentric right extension	0.93	NR	3.6	NR	9.9
		Concentric left extension	0.96	NR	3.8	NR	10.5
		Concentric right flexion	0.89	NR	4.9	NR	13.5
		Concentric left flexion	0.96	NR	4.8	NR	13.3
		Eccentric right flexion	0.91	NR	5.1	NR	14.1
		Eccentric left flexion	0.98	NR	3.5	NR	9.7
Biodex System 3, 2018 [23]	26	Concentric extension	0.99	NR	NR	NR	NR
		Concentric flexion	0.97	NR	NR	NR	NR
		Eccentric flexion	0.96	NR	NR	NR	NR
SMM iMoment, 2020	19	Concentric right extension	0.99	6.16	2.54	17.07	7.04
		Concentric left extension	0.97	10.67	3.85	29.57	10.67
		Concentric right flexion	0.96	6.66	4.54	18.45	12.58
		Concentric left flexion	0.98	4.91	3.47	13.60	9.63
		Eccentric right flexion	0.89	12.26	6.93	33.99	19.22
		Eccentric left flexion	0.95	8.28	4.93	22.95	13.67

ICC-intraclass correlation coefficient; SEM-standard error of measurement; SRD-smallest real difference; NR-not reported

Compared to other reports, we obtained lower SEM % values [5,7,14,19]. Our SEM % values were lowest for the right quadriceps peak concentric torque (2.54%) and the left hamstring concentric torque (3.47%), and the highest for hamstring eccentric peak torque (4.93–6.93%). Slightly higher SEM % values, ranging from 3.50% to 10.71%, were reported by previous studies [5,7,14,19]. Interestingly, we noted an intra-dynamometer reliability differences using short (30°) or full ROM (70°) on the REV 9000 dynamometer [7,19]. The full ROM demonstrated higher absolute reliability (3.5–5.1%) [7] compared to short ROM (8.52–10.71%) [19]. In this case, the number of maximal repetitions in the measurement might play a possible role, as five maximal repetitions [7] using full ROM may induce higher reliability compared to only two maximal efforts using short ROM protocol [28]. Additionally, our results also showed low to moderate error (SRD %) to detect a real test-retest change, which is supported by previous studies. The SRD % in other studies was very similar [5,7,14], ranging from 9.7% [7] to 19.47% [5]. One possible reason for such minor discrepancies may lie in the protocol design, which consisted of five maximal repetitions with longer rest duration (2 minutes) between sets, while others mainly used two to three maximal effort separated by 60 seconds break [5,14]. Shorter breaks between testing sets may lead to a higher rate of fatigue accumulation and subsequent performance decrement in the later stages of the given protocol. Moreover, there were no differences between longer test-retest break (>7 days) in our case compared with shorter test-retest breaks (96 h) reported in other studies [7,14]. For example, Sole et al. (2007) conducted a second measurement after 7 days and reported the highest values of SRD % to date using the Kincom 500H dynamometer. Also, there was no heteroscedasticity observed in either of the measurement via Bland-Altman plots or correlation, which is comparable with previous studies [5,19].

Lastly, we must also emphasize some mechanical characteristics of this new dynamometer that could additionally influence good-to-excellent reproducibility parameters. First, the chair has a very stable backrest and the movement of the chair was motorized, allowing us to align dynamometer and knee joint axes very precisely. The majority of other commercially available dynamometers have manual adjustment of the chair and increments for adjustments are not as smooth as in the present case. Other dynamometer characteristics, such as maximal torque on the rotational axis with maximal angular velocity were comparable to other devices.

During the course of our study we have identified few limitations. Firstly, we have included only young, physically active adults, which means that our findings may not be valid for different age groups or in groups of patients. Thus, it would be interesting to extend our research to include such specific groups. Secondly, our study would benefit from cross confirmation of our strength findings with commercially available dynamometers (e.g., Biodex or CSMI Norm) [2,9], however, this comparison was not possible at the time of the study. Finally, future reliability studies should implement the same leg testing order at test and rest for each participant to minimize potential onset of central fatigue [29], which was not observed in our study.

In conclusion, our study has established moderate to excellent reliability and reproducibility with low measurement error for knee flexors and extensor using a novel self-constructed SMM iMoment isokinetic dynamometer. Therefore, we believe that our results indicate the potential applicability of the SMM iMoment isokinetic dynamometer in research, sport rehabilitation and exercise settings in order to monitor athlete progress during training. Nevertheless, future research is needed to assess the reliability of unilateral and bilateral imbalance muscle ratios and the reliability of SMM iMoment dynamometer in clinical settings.

## Supporting information

S1 Data (SAV)
